# Exploratory Analysis Using Deep Learning for Water-Body Segmentation of Peru’s High-Mountain Remote Sensing Images

**DOI:** 10.3390/s24165177

**Published:** 2024-08-10

**Authors:** William Isaac Perez-Torres, Diego Armando Uman-Flores, Andres Benjamin Quispe-Quispe, Facundo Palomino-Quispe, Emili Bezerra, Quefren Leher, Thuanne Paixão, Ana Beatriz Alvarez

**Affiliations:** 1 LIECAR Laboratory, Universidad Nacional de San Antonio Abad del Cusco (UNSAAC), Cusco 08003, Peru; daufslash102@gmail.com (D.A.U.-F.); benja.msl.321@gmail.com (A.B.Q.-Q.); facundo.palomino@unsaac.edu.pe (F.P.-Q.); 2 PAVIC Laboratory, University of Acre (UFAC), Rio Branco 69915-900, Brazil; emili.bezerra@sou.ufac.br (E.B.); quefren.leher@sou.ufac.br (Q.L.); thuannepaixao@gmail.com (T.P.); ana.alvarez@ufac.br (A.B.A.)

**Keywords:** deep learning, satellite imagery, remote sensing, water body segmentation, high-mountain ecosystem, Peru

## Abstract

High-mountain water bodies represent critical components of their ecosystems, serving as vital freshwater reservoirs, environmental regulators, and sentinels of climate change. To understand the environmental dynamics of these regions, comprehensive analyses of lakes across spatial and temporal scales are necessary. While remote sensing offers a powerful tool for lake monitoring, applications in high-mountain terrain present unique challenges. The Ancash and Cuzco regions of the Peruvian Andes exemplify these challenges. These regions harbor numerous high-mountain lakes, which are crucial for fresh water supply and environmental regulation. This paper presents an exploratory examination of remote sensing techniques for lake monitoring in the Ancash and Cuzco regions of the Peruvian Andes. The study compares three deep learning models for lake segmentation: the well-established DeepWaterMapV2 and WatNet models and the adapted WaterSegDiff model, which is based on a combination of diffusion and transformation mechanisms specifically conditioned for lake segmentation. In addition, the Normalized Difference Water Index (NDWI) with Otsu thresholding is used for comparison purposes. To capture lakes across these regions, a new dataset was created with Landsat-8 multispectral imagery (bands 2–7) from 2013 to 2023. Quantitative and qualitative analyses were performed using metrics such as Mean Intersection over Union (MIoU), Pixel Accuracy (PA), and F1 Score. The results achieved indicate equivalent performance of DeepWaterMapV2 and WatNet encoder–decoder architectures, achieving adequate lake segmentation despite the challenging geographical and atmospheric conditions inherent in high-mountain environments. In the qualitative analysis, the behavior of the WaterSegDiff model was considered promising for the proposed application. Considering that WatNet is less computationally complex, with 3.4 million parameters, this architecture becomes the most pertinent to implement. Additionally, a detailed temporal analysis of Lake Singrenacocha in the Vilcanota Mountains was conducted, pointing out the more significant behavior of the WatNet model.

## 1. Introduction

The growing concern about climate change effects has led to the identification of certain ecosystems as indicators of environmental impacts. Lakes are sensitive and rapid sentinels of climatic and hydrological changes in catchments [1], constituting valuable tools to understand environmental dynamics. Water is a vital resource given its fundamental role in societies and ecosystems, especially in high-mountain ecosystems such as the Andes [2]. The high-mountain ecosystem serves as a vital freshwater reservoir, benefiting both the mountainous terrain and downstream areas because of the presence of glaciers, snowcaps, rivers, wetlands, and lakes in these regions [3]. The Peruvian Andes contain 70% of the world’s tropical glaciers, covering an area of more than 1600 km^2^ [4]. However, these glaciers have had significant retreat in recent decades, generating spatial and temporal changes in both ice and water bodies, and these changes can economically affect the population in aspects such as agriculture, access to drinking water, electricity generation [5], maintenance of ecosystems, and tourist activities [6]. In addition, they are associated with Glacial Lake Outburst Flood (GLOF) disasters. In this scenario, there is a need to develop automated and efficient methods for precise lake sampling in high-mountain environments to address the challenges of climate change in these ecosystems, prevent and mitigate disasters, and adequately manage and protect water resources [7].

The use of remote sensing sensors has become an important source of information for Earth observation [8], especially high-optical-resolution satellite sensors with high spatial and spectral resolution. The use of this type of imagery has become the main technical means to obtain dynamic information on bodies of water because these images have the characteristics of short update time, extensive land coverage, few restrictions, and the possibility of acquisition in real time [9]. Despite great advances in the field of water-body segmentation, this is still a challenging task due to various characteristics of these bodies, such as their irregular shapes and varying sizes, as well as the variability of the spectral reflectance of the different bands due to meteorological conditions, capture angles, clouds, and noise [10]. Adding to the complexity is the interaction of elements such as vegetation, infrastructure, and climatic conditions, which often require manual operations to interpret the imagery [11].

Every year, numerous studies analyze water bodies. Within this field, studies that use lake segmentation can be divided into two groups: (a) methods based on spectral analysis of indices and (b) classification methods based on machine learning (ML) [12]. The spectral-based methods focus on water index thresholding techniques, such as the Normalized Difference Water Index (NDWI) [13] and Modified Normalized Difference Water Index (MNDWI) [14], which use different combined bands to obtain a specific spectral response that highlights the water bodies compared to the background. Synthetic-Aperture Radar (SAR) imagery is also used, which has the advantage of being resistant to all types of weather, providing data both day and night, and not being affected by clouds. To achieve segmentation with this approach, the Sentinel-1 Dual Polarization Water Index (SDWI) [15] is used. The thresholding of these indices for the accurate detection of water bodies represents a large amount of work, low automation, and low efficiency [12] since the classification thresholds must be set manually for each scenario. Although this approach shows high accuracy in extracting water from satellite imagery, the aforementioned drawbacks do not allow monitoring with high temporal frequency. To address these difficulties, adaptive thresholding algorithms such as Otsu [16] have been used to segment water bodies. Although this approach is semi-automatic, it is limited in its global applicability.

On the other hand, ML-based methods try to establish a relationship between water bodies in satellite images and their respective masks. Unsupervised learning algorithms have been developed to address the problem of water body segmentation from satellite images, utilizing techniques such as K-means clustering [17] and probabilistic models like Markov Random Fields (MRFs) [18]. In the field of supervised learning, algorithms based on Support Vector Machines (SVMs) [19] and Random Forests (RFs) [20] have been developed for remote sensing (RS) image segmentation. However, these traditional ML methods have difficulties because they rely only on individual pixels to perform segmentation, so they have low precision and are difficult to apply to large images [21]. These traditional methods for water body extraction have difficulties in accurately detecting the boundaries of water bodies, especially in complex scenarios, which makes their automatic application difficult in any geographical environment [22]. Given the limitations of the mentioned approaches to water body segmentation, different models based on deep learning have been fundamental in semantic image segmentation, especially with the introduction of Convolutional Neural Networks (CNNs) [23]. Architectures such as Fully Convolutional Networks (FCNs) [24] and encoder–decoder networks like UNet [25] have been developed, all achieving good results in various fields. Other architectures like PSPNet [26] and Deeplab [27] use techniques such as spatial pyramids and dilated convolutions to capture both global and local contexts. Despite the efficacy of these architectures in other contexts, their direct application to water-body detection presents issues such as low precision and blurry edges, especially in large satellite images.

It has been shown that the implementation of deep learning models designed specifically for water-body segmentation achieves superior results with the use of deep neural networks and multispectral satellite data. Several studies have approached similar challenges to those proposed in this research, especially in the high-mountain regions of the Himalayas, which have complex terrain features. The research by Wang et al. [28], Thati and Ari [29], and Zhao et al. [30] provides a basis for lake segmentation in very challenging environments. These studies emphasize the importance of using multispectral information and innovative approaches to effectively manage the complexities of high-mountain terrain. In addition, comparative analyses of the results with deep learning techniques and classical water body segmentation methods are highlighted.

Specifically, in the field of lake segmentation, two architectures stand out: WatNet and DeepWaterMapV2. WatNet, proposed by Luo et al. [31], is a hybrid model with the MobilenetV2 architecture [32] as the backbone for the encoder and ASPP and DeepLabV3+ [27] as the decoder. This combination results in a lightweight model capable of accurately segmenting water bodies in both urban and mountain environments, achieving accuracies of over 95% in validations with Sentinel-2 images. Due to its excellent performance, WatNet will be used in this research for water-body segmentation in high-mountain conditions. Furthermore, DeepWaterMapV2 by Isikdogan et al. [33], trained on Landsat-8 images, is capable of making predictions on Landsat 5, 7, and 8 images, as well as Sentinel-2. This model was chosen for its robustness and resistance to various environmental conditions, noise, and clouds, making it particularly suitable for this study.

On the other hand, recent advances in medical image segmentation combine probabilistic diffusion models and transformers. MedSegDiffV2 [34] exemplifies this approach by integrating UNet with transformers through anchoring and semantic conditioning through the Spectral Space transformer; this architecture obtains excellent segmentation results in its field.

Therefore, this paper presents an exploratory analysis of the Peruvian Andes focused on monitoring lakes with high temporal and spatial frequency. Andean lakes are essential for mountain ecosystems, supplying water, promoting biodiversity, and supporting various human activities. Accurate and up-to-date mapping of these lakes is crucial for environmental monitoring, water resource management, and sustainable development planning. To address the segmentation problem of lakes in complex mountain environments, we explored the capabilities of the WaterSegDiff model, a modified version of MedSegDiffV2 designed to process multispectral images, along with well-established methodologies in lake segmentation tasks: NDWI with Otsu thresholding, DeepWaterMapV2, and WatNet. The accuracy of the results obtained by each method was evaluated using the metrics MIoU (Mean Intersection over Union), Pixel Accuracy, and F1 Score, widely used in the field of semantic segmentation.

In summary, the contributions of this research are:

- Using Landsat-8 multispectral images, a comprehensive dataset of high-mountain lakes in the Peruvian Andes has been created, expanding knowledge of surface waters in this region. This dataset is divided into a training, validation and test.
- This study explores the behavior and performance of WaterSegDiff, a diffusion model with transformers, for remote sensing lake segmentation in complex high-mountain environments and compares WaterSegDiff with established methods such as NDWI, WatNet and DeepWaterMapV2.
- Temporal analysis of Lake Singrenacocha (Vilcanota Mountains, Peru) for 2014, 2016, 2018, and 2020 using segmentation techniques to understand the impact of environmental conditions and evaluate the practical usefulness of the models in real-world challenges.

This paper is structured as follows: Section 2 covers related work. Section 3 details the study area, data preprocessing, and description of the materials and methods. Section 4 presents the experiments and results. Section 5 discusses the findings. Finally, Section 6 describes the conclusions and outlines future studies.

## 2. Related Work

With the development of computer science and the large amount of information provided by satellites, DL-based methods have gained prominence in the field of semantic segmentation using high-resolution satellite imagery. One of the models used in this context was proposed by Li et al. [35], who developed a Fully Convolutional Network (FCN) for water body segmentation in urban areas. The model was trained and evaluated using images from the Gaofen-2 satellite, which provides high-resolution images in four bands: blue, red, green, and near-infrared, which were cut into patches of 256 × 256 pixels. The study evaluated the performance impact of different configurations, such as input data, data augmentation, and the use of pretrained networks. Some geographical and atmospheric features, such as the presence of vapor and haze, as well as differences in shadow characteristics of mountains and buildings, limit the model’s generalization.

Liu et al. [36] implemented a variant of UNet based on the standard encoder and decoder architecture. However, in this version, the skip connections between them are made differently. Information from the contracting part is passed through a multi-scale pyramid pooling module (MSPP) inspired by DeepLabV3+. For training and evaluating the results, they used data from the Gaofen 2020 Challenge, which consists of RGB images, along with images from Landsat-8. The variability in the shape and size of water bodies, as well as the presence of clouds, are considered limitations in the model’s predictions. Additionally, the availability of suitable datasets is noted as crucial for improving performance.

Chen et al. [12] proposed a hybrid model based on the combination of K-Net with other semantic segmentation models, including FCN, DeeplabV3, PSPNet, and UperNet. These models were validated using the 2020 Google Tibetan Plateau Lakes dataset, which comprises RGB images cropped into 256 × 256 patches, achieving remarkable segmentation accuracy. However, a limitation of the model is its inability to leverage information from other spectral bands.

Kadhim and Premaratne [37] proposed a new architecture for water body segmentation, based on the integration of residual convolutional models, attention gates, and supervised deep learning. The differential in this research is the use of real-world satellite images in RGB, with the dataset composed of public Sentinel-2 images containing only the three RGB bands. After pre-processing, the final dataset contains 2686 images, adjusted to a size of 256 × 256.

Recently, the application of probabilistic diffusion models and Transformer models has emerged as a promising alternative. Inspired by recent advances in natural language processing [38], computer vision [39], and image synthesis from Gaussian noise [40], these models offer the capability to capture complex spatial and contextual relationships and generate high-quality images, making them suitable for segmentation tasks. Although diffusion models have been primarily used in image generation [41,42], as well as for inpainting problems [43,44], they have recently been used for semantic segmentation problems [45,46]. Baranchuk et al. [45] demonstrate that probabilistic diffusion models (DDPMs) offer superior generative quality in the task of semantic segmentation with few samples. Tan et al. [47] proposed a semantic diffusion network (SDN) which includes a parameterized semantic difference convolution operator followed by a feature fusion module. Since diffusion models produce segmentation masks from random noise, it is necessary to condition the model using the original image as a reference [48]. Based on [49], Wu et al. present the MedSegDiffV2 [34], an architecture that effectively integrates the UNet model with transformers through the implementation of anchor conditioning and semantic conditioning based on the Spectral-Space transformer (SS-Former), obtaining excellent results in the field of medical imaging.

Inspired by related research, this study processes Landsat-8 satellite images to create 256 × 256 image patches. The pre-processing of the images and generation of the dataset follows the methodology proposed by Bezerra et al. [50] in which a dataset is generated with Landsat-8 multispectral images.

The research described in this paper includes a comparison of the WatNet, DeepWaterMapV2 and WaterSegDiff models, the last one based on MedSegDiffV2, to address the limitations identified in complex environments such as the Andean regions.

## 3. Materials and Methods

This section includes a comprehensive description of the geographical location of the area studied in this research, the data acquisition process, the methodology used to pre-process the data and generate the dataset, and the process used to create the masks. In addition, the NDWI methodology with Otsu thresholding, WatNet, and DeepWaterMapV2 will be described and the WaterSegDiff model will be presented in detail. Finally, the metrics used to analyze the performance of the methodologies are described.

### 3.1. Study Area

**Figure 1:**
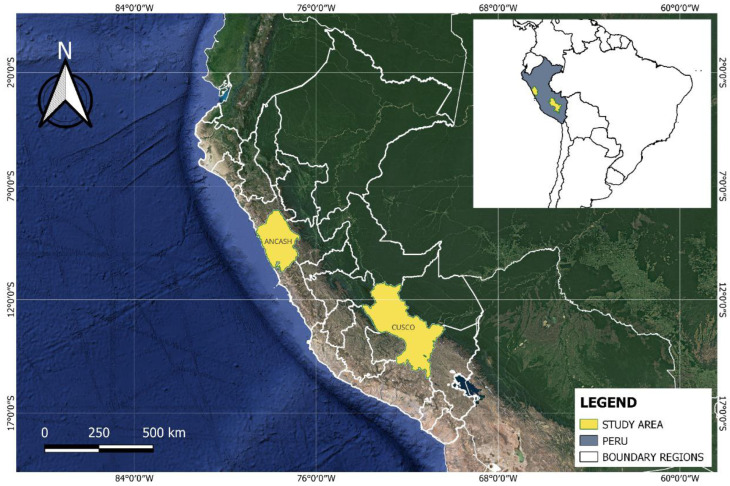
Location of the study area.

This study focus on the lakes located in the Peruvian Andes, specifically in the regions of Cusco and Ancash. These areas were chosen because they contain the highest concentration of lakes in the country and play an important role in the ecological and environmental processes of the Andean region. The department of Ancash is located in the central Andes, and Cusco is in the southeastern Andes of Peru, as shown in Figure 1.

Ancash has an area of 39,915 km^2^, which represents 2.8% of the Peruvian territory. It concentrates a population of 1,083,519 inhabitants (INEI, https://www.gob.pe/inei/, accessed on 24 January 2024). This region has 937 lakes of glacial origin, covering an area of 59.88 km^2^, most of them located between 4000 and 5000 m above sea level. Furthermore, Cusco extends over an area of 71,987 km^2^, which represents 5.6% of the Peruvian territory. It concentrates 1,205,527 inhabitants (INEI, https://www.gob.pe/inei/, accessed on 24 January 2024). Cusco has 1288 lakes of glacial origin, being the second region in Peru with the largest area of water bodies, with a surface of 166.95 km^2^. Most of these lakes are located between 4500 and 5000 m above sea level [51].

### 3.2. Dataset

#### 3.2.1. Data Acquisition

**Figure 2:**
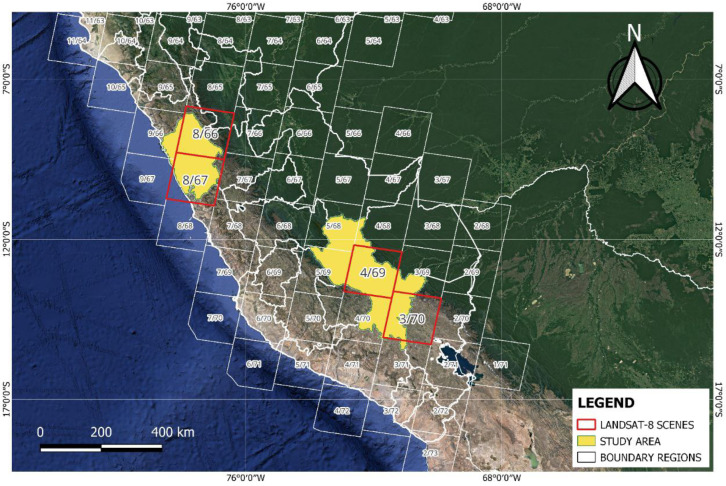
Landsat-8 scenes selected for study.

The scenes selected were acquired between the years 2013 and 2023 and cover the largest number of lakes in the study area. Each scene has a resolution of 7800 × 7600 pixels and covers an area of 185 km by 180 km. Figure 2 shows in red the scenes 008/066, 008/067, 004/069, and 003/070, which are used to prepare the dataset.

A total of 39 scenes were used to compose the dataset. The scenes were selected between the months of May and September, which is the rainless season in Peru. A maximum cloud cover of 10% per scene was considered in order to adequately observe the water bodies.

The satellite image dataset was obtained from the Landsat-8 satellite through the U.S. Geological Survey (USGS) Earth Explorer. Based in [52], Landsat-8 is equipped with the Operational Land Imager (OLI) sensor, which captures data across nine spectrally distinct bands, offering enhanced radiometric accuracy thanks to its 16-bit dynamic range and spatial resolution ranging from 15 to 30 m. The Landsat-8 mission provides products with varying levels of processing. This study uses data from the Level 1 Terrain Precision Correction (L1TP) collection, which is 16-bit unsigned and can represent up to 65,536 gray levels. These data can be rescaled again to obtain Top of Atmosphere (TOA) reflectance and/or radiance values using the radiometric rescaling coefficients included in the metadata. L1TP products are radiometrically calibrated and orthorectified using ground control points (GCPs) and digital elevation models (DEMs) to correct for displacement due to relief.

#### 3.2.2. Data Preprocessing

**Figure 3:**
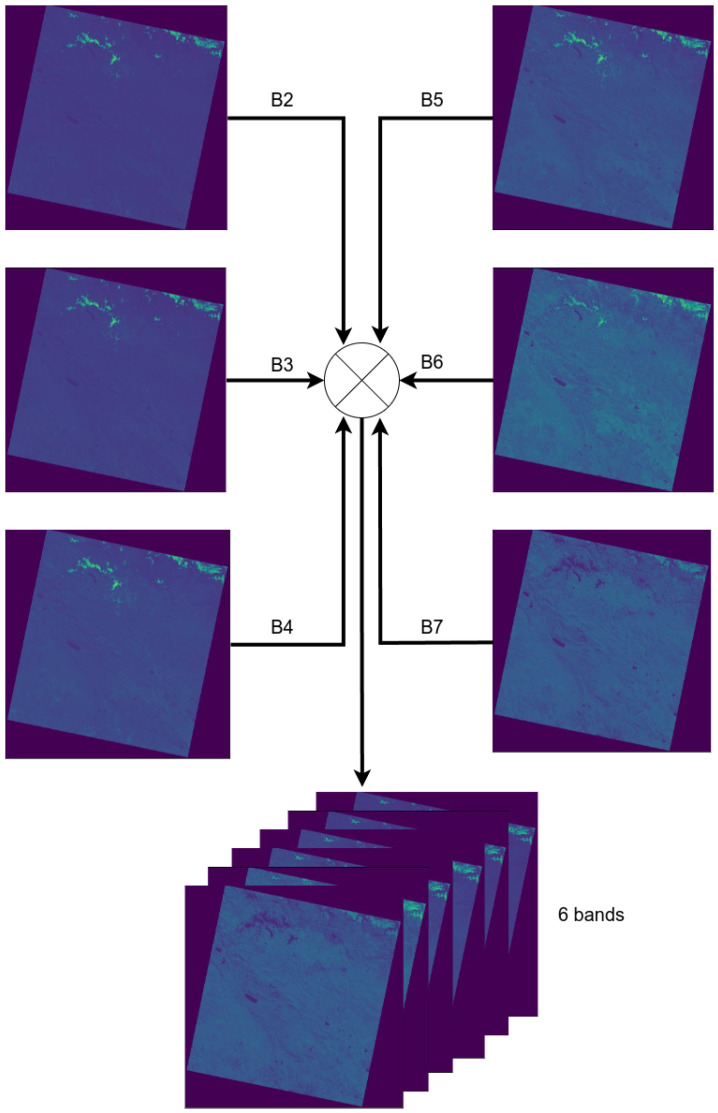
Combining process from B2 to B7 into a single 6-channel image.

The 6-band stacking of the Landsat-8 OLI sensor was performed individually on each of the 39 selected scenes, using bands 2 to 7 as described in Table 1. This process resulted in a 6-channel image, as illustrated in Figure 3.

The generated 6-band image serves as a fundamental basis for creating the final dataset. These bands capture key multispectral characteristics of the scene, providing the necessary information for conducting the study.

**Figure 4:**
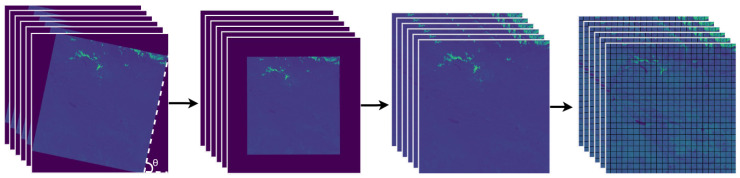
From left to right: (θ,ρ) parameter space, deskwed image, cropped image, and division of the image into 256 × 256 pixel patches.

To obtain the dataset, it is necessary to deskew the satellite images. This process is performed by a series of steps described in [50]. Deskewing involves determining the inclination angle θ obtained by the Hough transform, which is efficient for identifying collinear sets of points in images by mapping the points to a set of parameters (θ,ρ) that define a line in parameter space. The plane is limited from $\left[0,180^{\circ }\right]$ for θ and from [−R,R] for ρ (delimiting a rectangle that encompasses the region of interest in the quantization) and records the presence of collinear sets through a two-dimensional accumulator system [53], which allows calculating the inclination angle θ of the satellite image. The image is rotated based on the angle found. The Canny function [54] is then used to distinguish the edges of the data of interest from the areas without data. Based on the edges found, the areas without data are eliminated from the image and then divided into patches of 256 × 256 pixels to reduce the computational cost of training. This process is shown in Figure 4.

Of all the generated patches, only those containing lakes are selected. To do this, a visual inspection of the patches is performed, considering only the RGB images (B4, B3 and B2). As a result, the dataset is composed of 1344 patches of 6-band satellite images from the 39 selected scenes. To enhance data diversity during the training process of the models, the database was expanded to 4032 images through data augmentation using horizontal and vertical flipping.

Following the methodology proposed in [33], which also aimed to perform a lake segmentation, the dataset was split into 10% for validation, another 10% for testing, and the remaining 80% for training, as shown in Table 2. This split was randomized to ensure the representativeness of the resulting datasets. It is important to note that the performance analysis was conducted on the test subset.

#### 3.2.3. Masks

**Figure 5:**
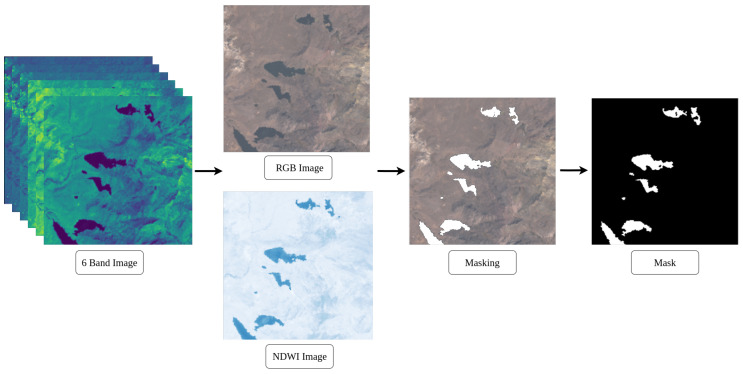
Mask creation process.

Making masks is a meticulous process that requires manual intervention. To establish fundamental truths, patches containing 6 multispectral bands are considered, from which their NDWI and RGB representations are extracted. This helps locate lakes in the 6-band image, aiding visual identification of lake areas. However, the NDWI is vulnerable to phenomena such as shadows and snow-covered areas, and RGB may not visualize certain areas easily. Therefore, to ensure accuracy in challenging terrain, each lake is located using the Google Earth Explorer tool, which offers high-resolution images for distinguishing shadows, glaciers, and lakes more effectively. As illustrated in the Figure 5, mask generation begins with the six-channel image. From there, the RGB channels are selected to visualize the lakes to be extracted.

The NDWI image provides visual support for the masking process, resulting in the final binarized mask. In this mask, the white pixels represent the lakes and the black pixels indicate the background.

### 3.3. NDWI

The Normalized Difference Water Index (NDWI) [13] is used to highlight water bodies in satellite imagery. It works by reducing the reflectance of vegetation and soil compared to the reflectance of water, allowing the presence and distribution of water on the Earth’s surface to be identified. The NDWI is obtained by calculating specific bands of the electromagnetic spectrum. For Landsat-8, the visible green band (B3) and the near-infrared band (B5) are used. These bands are selected because water tends to absorb radiation in the NIR and reflect it in the visible green, which produces a significant difference in the spectral response between areas with water and areas without water. The NDWI for Landsat-8 imagery is calculated using Equation (1).

(1) $$\mathrm{NDWI}=\frac{B3-B5}{B3+B5}$$

Currently, the NDWI index has several applications, such as water monitoring [55], water resource management [56], and aquatic ecosystem quality assessment [57]. In image processing of normalized indices such as NDWI, it is common to use the Otsu thresholding method to discriminate between different classes [58]. This method has the advantage of finding the optimal threshold value that maximizes the variance between two classes in an image. In this study, the NDWI is used in combination with the Otsu thresholding method for lake segmentation. The NDWI is first computed to highlight the water areas in the image. Then, the Otsu method is applied to automatically determine an optimal threshold that discriminates between pixels corresponding to water.

### 3.4. WatNet

**Figure 6:**
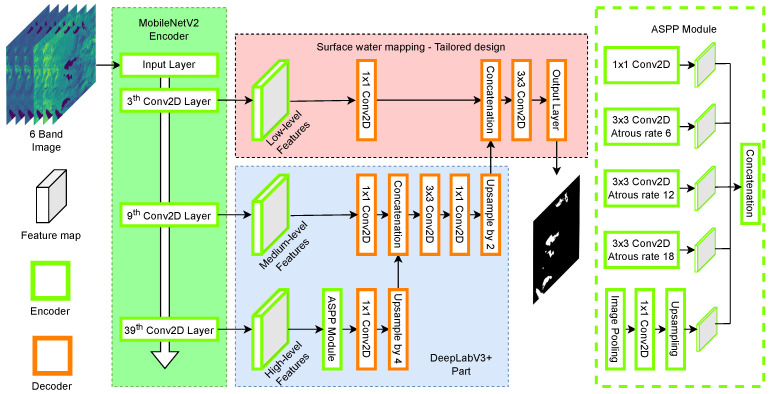
WatNet model architecture.

WatNet [31] is a deep convolutional neural network that employs an encoder–decoder structure for semantic segmentation of water surfaces using state-of-the-art deep classification models originally proposed for processing Sentinel-2 imagery. The architecture of the model is shown in Figure 6.

The encoder module is responsible for encoding the contextual information of an image into multiscale features, which are later decoded into pixel-based classification maps. WatNet considers the performance of the model in terms of accuracy and classification efficiency by selecting state-of-the-art lightweight models, specifically, MobileNetV2 [32] and DeepLabV3+ [59] as encoder and decoder modules, respectively. DeepLabV3+ implements Atrous Spatial Pyramid Pooling (ASPP) to capture features at multiple scales, its decoding module incorporates both high-level features, using upsampling at a scale of 4, and low-level features. This data is concatenated and fed into the decoder. These features are extracted from different levels of the MobileNetV2 encoder, providing diverse contextual information at multiple scales. The combination of these features allows generating a prediction map with high scale accuracy. With respect to the MobileNetV2 encoder, it is configured to process multispectral images composed of 6 bands. In addition, the number of original MobileNetV2 feature maps is reduced from 256 to 128 channels to obtain a lighter model.

### 3.5. DeepWaterMapV2

**Figure 7:**
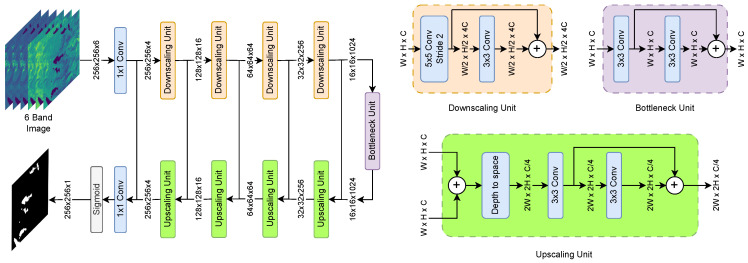
DeepWaterMapV2 model architecture based on 3 primary blocks.

DeepWaterMapV2 is also a deep convolutional neural network, and was developed by Isikdogan et al. [33], inspired by the Fully Convolutional Network (FCN) and U-Net architectures. Its design is based on three main components: downscaling, a bottleneck, and upscaling units. The downscaling units employ 5×5 convolutions with a stride of 2, followed by 3×3 residual convolutions to maintain the size of the feature map and reduce memory usage. The bottleneck unit consists of two residual convolutional layers, 3×3, without modifying the spatial resolution. Finally, upscaling units use a depth-to-space transformation followed by 3×3 residual convolutions to increase resolution. All layers include batch normalization and ReLU activation, except the first and last. The input and output of the network are 1×1 convolutional layers, with the output layer using a sigmoid activation to generate water pixel probabilities. The model architecture is shown in Figure 7.

DeepWaterMapV2 is designed to process large images efficiently because instead of processing a large number of channels in the first layers of the model, the largest number of trainable parameters is moved to the layers that process lower resolution feature maps, optimizing memory and efficiency. The model was developed to segment water bodies based on 6-band Landsat-8 imagery.

### 3.6. WaterSegDiff

**Figure 8:**
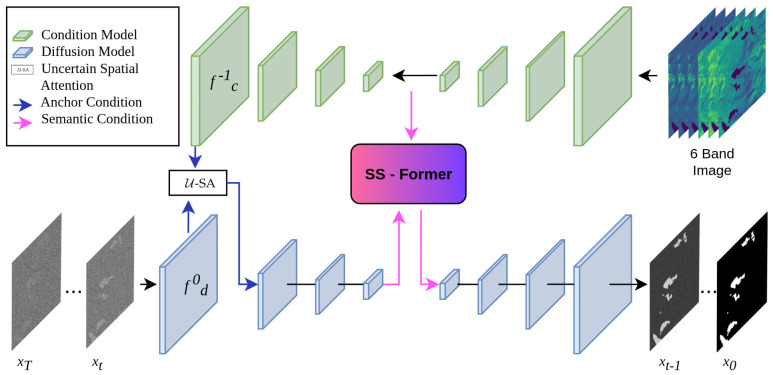
General architecture of WaterSegDiff based on a conditioning model and a diffusion model that integrate their information through two conditioning mechanisms, U-SA and SS-Former.

This research proposes WaterSegDiff, a model based on the work of Wu et al. [49], known as MedSegDiff-V2. This model employs a Diffusion Probabilistic model (DPM) to perform segmentation in a variety of medical image analysis tasks. MedSegDiff-V2 is a transformer-based diffusion framework that employs two different conditioning techniques, anchor and semantic conditioning. These techniques allow the effective integration of conditioning features into the diffusion model. To achieve this, the model uses two UNet architectures, one for the diffusion block and another for the conditioning block. The UNet conditioning block serves as a segmentation feature extractor from the raw original image, learning the most relevant features. The segmentation features are integrated with the noise mask information using the anchor conditioning technique, which implements the Uncertain Spatial Attention (U-SA) mechanism. The integrated data are then introduced to the encoder of the UNet Diffusion model. The semantic conditioning integrates the high-level characteristics obtained by both the diffusion and conditioning blocks through a transformer mechanism called Spectrum-Space transformer (SS-Former), which is a cross-attention mechanism in the frequency domain that allows aligning the noisy image information with the segmentation features of the raw image. Both conditioning mechanisms are used to address the compatibility issue of combining a UNet model with DPM, implementing an interface between both models that allows reducing the large variance in the transformer configuration. The architecture of WaterSegDiff is shown in Figure 8.

At each step *t* of the diffusion process, a noise mask $x_{t}$ is introduced to the UNet diffusion model. This model is conditioned using segmentation features extracted from the raw image through the UNet conditioning model. The diffusion process is conditioned using the anchor conditioning and semantic conditioning techniques. The former allows the diffusion model to be initialized with an approximate but static reference, which helps reduce variance in the diffusion. The latter technique, by using SS-Former, connects the noise and high-level segmentation information to introduce it into the decoder of the diffusion model, thereby generating a more robust representation that takes advantage of the global and dynamic nature of the transformer [60]. This conditioning that is introduced to the decoder of the diffusion model can be expressed as in Equation (2).

(2) $$\epsilon_{\theta }\left(x_{t},I,t\right)=D\left(TransF\left(E_{t}^{I},E_{t}^{x}\right),t\right),$$

where $E_{t}^{I}$ represents the high-level features of the raw image and $E_{t}^{x}$ represents the high-level features of the current noisy image. Using a transformer, both characteristics are incorporated and passed through the decoder *D* of the diffusion model.

#### 3.6.1. Anchor Conditioning

To improve the stability and accuracy in the prediction of the diffusion model, the anchor conditioning operation with U-SA is introduced. This operation integrates an approximate anchoring feature from the conditioning model into the diffusion model, providing it with a correct prediction range and allowing the results to be refined [49]. The U-SA mechanism is used to fuse features, representing the uncertainty nature of conditional features. For the U-SA application, the last layer of the conditioning model $f_{c}^{-1}$ is integrated with the first layer of the diffusion model $f_{d}^{0}$, it can be expressed as in Equations (3) and (4).

(3) $$f_{anc}=Max\left(f_{c}^{-1}*k_{Gauss},f_{c}^{-1}\right),$$

(4) $$f_{d}^{\prime 0}=Sigmoid\left(f_{anc}*k_{Conv_{1\times 1}}\right)\cdot f_{d}^{0}+f_{d}^{0}$$

In practical terms, a Gaussian kernel is applied to the final features of the conditioning model to obtain smooth activation, and to improve accuracy, the maximum value between the smoothed mapping and the original feature map is taken. The feature channels are then reduced to 1 by using a 1×1 convolution, in a similar way to the spatial attention implementation [61], a Sigmoid activation is applied, multiplied by $f_{d}^{0}$ and added to each channel of $f_{d}^{0}$.

#### 3.6.2. Semantic Conditioning with SS-Former

**Figure 9:**
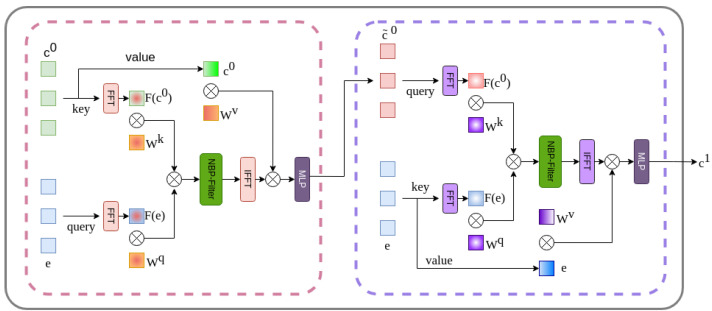
SS-Former internal architecture consisting of two symmetrical cross-attention modules.

To address the gap between diffusion model and conditional semantic embeddings, which affects the performance in matrix manipulations, the Spectrum-Space transformer (SS-Former) is used [49]. This approach employs a filter designated as the Neural Band Pass Filter (NBP-Filter) to align conditional semantic and diffusion noise features in the frequency domain. An SS-Former block consists of two symmetrical cross-attention modules, as illustrated in Figure 9.

The first encodes the diffusion embeddings into the semantic conditioning embeddings, while the next module encodes the previous result into the diffusion model embedding. In order to achieve this encoding, the deepest embedding feature of the conditioning model $c^{0}$ and that of the diffusion model *e* are taken into account, which are the inputs of the SS-Former block. The initial step is to transfer the inputs to Fourier space, designated by $F(c^{0})$ and F(e), respectively. In accordance with the vision transformer methodology, the feature map is separated into patches and projected linearly. Taking the input F(e) as query and $F(c^{0})$ as key, the affinity weight matrix M is calculated, as shown in Equation (5).

(5) $$M=\left(F\left(c^{0}\right)W^{q}\right){\left(F\left(e\right)W^{k}\right)}^{T},$$

where $W^{q}$ and $W^{k}$ are the learnable weights of query and key in Fourier space.

In order to map each value of M, which represents a specific frequency, to a continuous range of frequencies, a learnable neural network is applied, called Neural Band Pass Filter (NBP-Filter). This receives as input a coordinate map that is passed through a series of convolutional blocks and intermediate normalization layers to generate an attention map that will serve as a filter that will project the feature map to frequency magnitudes. The NBP-Filter is conditioned by using two MLPs (Multilayer Perceptrons) that embed the timestep information into two values that represent the mean and variance, values that are used to shift and scale the normalized features. Subsequently, the filter is multiplied element by element with M. The inverse Fourier transform is applied to the filtered affinity map $M^{\prime }$ and multiplied by the product of the conditional characteristics with $W^{v}$, which represents the learnable weights of value. This results in the equation shown in Equation (6).

(6) $$f=F^{-1}\left(M^{\prime }\right)\left(c^{0}W^{v}\right)$$

Finally, an MLP is applied to refine the attention characteristics, resulting in ${\tilde{c}}^{0}$, which serves as the input query in the subsequent symmetric attention block. Meanwhile, *e* is utilized as the key and value, integrating the segmentation characteristics within the noise domain. The output $c^{1}$ serves as an input conditioning embedding for the subsequent block.

#### 3.6.3. Loss Function

The loss function utilized for training the model is a combination of the standard noise prediction loss $L_{n}$ [40] and the anchor loss $L_{anc}$. The latter is defined as the weighted combination of Dice loss $L_{dice}$ and cross-entropy loss $L_{ce}$. The total loss function is represented in Equation (7).

(7) $$L_{total}^{t}=L_{n}^{t}+\left(t\equiv 0\left(mod\alpha \right)\right)\left(L_{dice}+\beta L_{ce}\right),$$

where t≡0(modα) controls the supervision times over the conditioning model through the hyperparameter α, and the cross entropy loss is weighted by the hyperparameter β, set to 5 and 10 respectively.

### 3.7. Evaluation Metrics

The Mean Intersection over Union (MIoU) [62] is defined by Equation (8) and is used as an evaluation metric in segmentation applications.

(8) $$\mathrm{MIoU}=\frac{1}{k+1}\sum_{i=0}^{k}\frac{\mathrm{TP}}{\mathrm{TP}+\mathrm{FP}+\mathrm{FN}}$$

Pixel Accuracy (PA) [63] is defined by the Equation (9), which measures the number of pixels that are classified as correct in the image. This value is calculated by dividing the number of pixels classified as correct by the total number of pixels in the image.

(9) $$\mathrm{PA}=\frac{\mathrm{TP}+\mathrm{TN}}{\mathrm{TP}+\mathrm{TN}+\mathrm{FP}+\mathrm{FN}}$$

The F1 Score [63] is defined by Equation (12) and is a metric commonly used to evaluate the quality of image segmentation. It is calculated using the Precision (Equation (10)) and recall (Equation (11)) metrics.

(10) $$\mathrm{Precision}=\frac{\mathrm{TP}}{\mathrm{TP}+\mathrm{FP}}$$

(11) $$\mathrm{Recall}=\frac{\mathrm{TP}}{\mathrm{TP}+\mathrm{FN}}$$

(12) $$F1\;\mathrm{Score}=\frac{2\times \mathrm{Precision}\times \mathrm{Recall}}{\mathrm{Precision}+\mathrm{Recall}}$$

where true positives are represented by TP, true negatives by TN, false positives by FP and false negatives by FN. These values are from the confusion matrix that compares the lake masks with their true values [36].

The high MIoU indicates that the model has a significant overlap between the predicted and true segmentation, while the high PA reflects the accuracy in identifying pixels belonging to their respective classes. A high F1 Score value indicates that the model has a good balance between precision and recall; i.e., it indicates a good overall performance of the model in identifying lakes.

## 4. Experiments and Results

This section describes the experiments carried out and presents the results achieved and analysis of the segmentation models used in the research. In Section 4.1 the implementation process is detailed, and in Section 4.2, the performance evaluation processes are described and qualitative and quantitative results are presented with their respective analysis. In Section 4.3, a temporal analysis of Lake Singrenacocha is carried out and segmentation results in diverse and challenging environmental conditions are compared.

### 4.1. Implementation Details

The quantitative and qualitative performance evaluation was carried out using the subset of tests described in Section 3.2.2. As a method of comparison, NDWI calculation was performed on the images using the green and near-infrared (NIR) bands (bands 3 and 5 after radiometric rescaling) to assess the presence and distribution of water in the areas of interest. To produce a water mask based on NDWI data, following the research approach of Wang et al. [9], Otsu’s method is employed for thresholding, automatically determining the optimal threshold value to separate water from other features in the image.

Moreover, comparative experiments with the WatNet and DeepWaterMapV2 models were performed using Tensorflow 2.15.0 as the framework on a Debian GNU/Linux 12 Bookworm system. This environment was run on an Intel Xeon^®^ W-2123 processor (Intel, Santa Clara, CA, USA) and an NVIDIA Quadro P2000 GPU (NVIDIA, Santa Clara, CA, USA) with 5 GB of memory. For the training of both models, BinaryCrossEntropy was used as the loss function and Adam as the optimizer, with a learning rate of 0.002 based in [31], a batch size of four, and an input size configured at 256 × 256 pixels. The models were trained for 150 epochs.

In contrast, all experiments with WaterSegDiff were performed using the PyTorch 2.0.1 framework on Ubuntu 22.04.3 LTS. The model was trained and tested on an Intel Xeon^®^ E5-2699 v3 (Intel, Santa Clara, CA, USA) and a single NVIDIA RTX 3090 Ti (NVIDIA, Santa Clara, CA, USA) with 24 GB. We employed a batch size of 5, and learning rate of $1\;\times \;10^{-4}$ during training. In accordance with the categorization proposed in [64], the hyperparameters were divided into three groups: diffusion process, model architecture, and training flags. Consequently, the hyperparameters align with the configurations outlined in [34]. In the inference process, 1000 diffusion steps and 15 sampling steps are used.

### 4.2. Performance Evaluation

The analyzed models were subjected to a comprehensive evaluation covering both quantitative and qualitative aspects. Initially, quantitative analyses were performed using the metrics (described in the Section 3.7) to provide an objective assessment of the models. Additionally, qualitative analyses were conducted to examine the visual and comparative performance of the models. These procedures enabled the measurement of accuracy, efficiency, and practical utility in addressing real-world challenges.

#### 4.2.1. Quantitative Analysis

All models were quantitatively validated using 402 test images, assessing accuracy through MIoU, PA, and F1 Score metrics for comprehensive segmentation quality evaluation. Experiments on a pre-processed dataset compared three deep learning models for lake segmentation (Table 3): NDWI-based with Otsu’s method, WatNet (a lightweight encoder–decoder for water segmentation), DeepWaterMapV2 (FCN with U-Net for water mapping), and WaterSegDiff (a transformer-based diffusion framework).

WatNet and DeepWaterMapV2 reached maximum performance at epochs 76 and 114, respectively. WaterSegDiff, which uses diffusion and transformers, lacks direct epoch equivalence, but due to its approach and volume of parameters, it turned out to be the most computationally demanding.

In terms of quantitative results (MIoU, Pixel Accuracy (PA), and F1 Score, described in Table 3), NDWI values represent the lowest results observed in all metrics. On the other hand, the values obtained by WatNet significantly outperform the NDWI methodology. DeepWaterMapV2 obtained the best results in the evaluation metrics. However, WaterSegDiff showed a competitive performance.

#### 4.2.2. Qualitative Analysis

**Figure 10:**
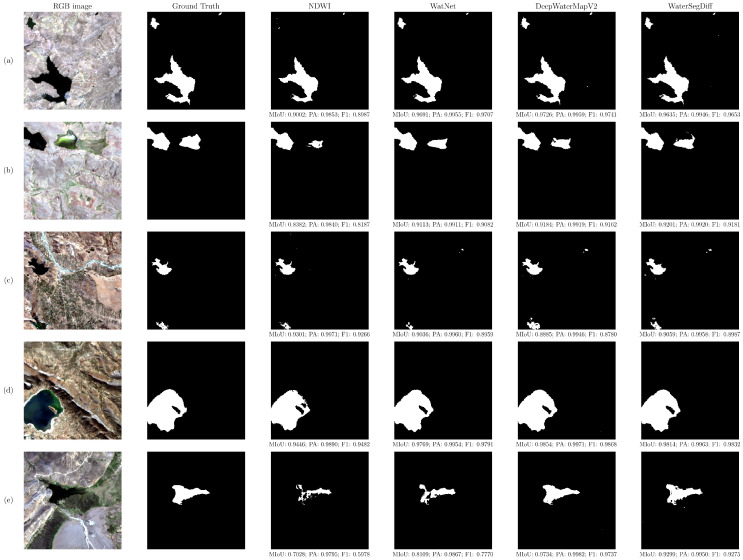
Qualitative analysis of 5 selected samples that represent large lakes with compact structures. Showing the RGB image, ground truth, NDWI, WatNet, DeepWaterMapV2, and WaterSegDiff results. (**a**) Large and irregular lake, (**b**) two lakes with compact structure, (**c**) scene with river crossing, (**d**) large lake in mountainous region, (**e**) lake surrounded by dense vegetation.

**Figure 11:**
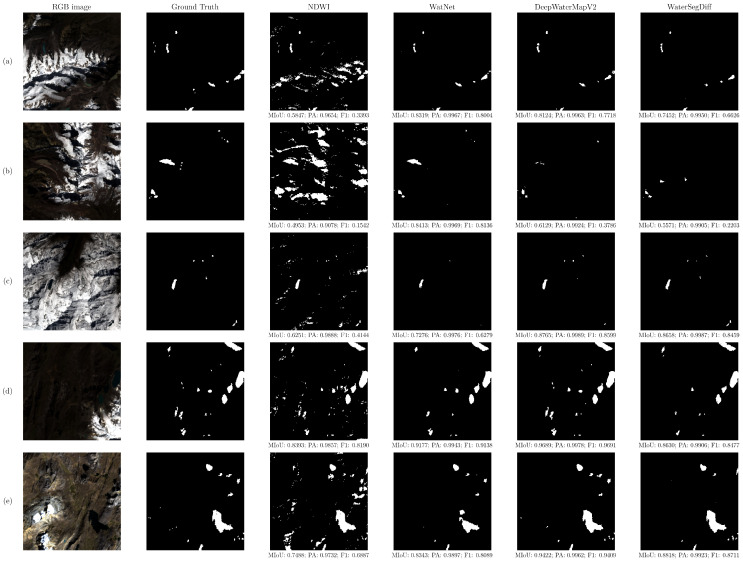
Qualitative analysis of 5 selected samples that represent small and dispersed lakes. Showing the RGB image, ground truth, NDWI, WatNet, DeepWaterMapV2, and WaterSegDiff results. (**a**,**b**) Snowy scene with shadows with presence of clear and turbid lakes, (**c**) completely snowy scene, (**d**,**e**) partially snowy area with scattered lakes.

A qualitative analysis was conducted by selecting 10 samples from the test set, divided into two groups to illustrate the differences between the methodologies explored. Figure 10 shows lakes with compact and large structures, meanwhile Figure 11 presents lakes that are dispersed and small.

The samples were selected to represent a range of lake sizes and complexities, allowing for a more comprehensive assessment. The images contain the segmentations using the NDWI with thresholding by Otsu, as well as the predictions of the WatNet, DeepWaterMapV2, and WaterSegDiff models, to observe the differences between the methodologies.

Figure 10a illustrates an irregularly shaped lake, characterized by its dark coloration in contrast to its mountainous and barren surroundings. The sample exhibits a minimal amount of shadowing and a relatively flat topography. It is observed that all methodologies display segmentations nearing to the ground truth when segmenting large lakes with a contrasting color compared to their surroundings. The NDWI methodology with Otsu thresholding obtains the poorest segmentation, encountering difficulties in detecting small lakes. On the other hand, WatNet has achieved outstanding results despite having difficulties in detecting sharp edges. In contrast, for this sample, DeepWaterMapV2 behaves as the most effective method in segmenting lakes. WaterSegDiff has obtained the second-best result, standing out for its ability to accurately identify the edges of large lakes.

In Figure 10b, two lakes with a compact structure are observed. The first of the two lakes, situated in the upper left, exhibits a dark coloration that is characteristic of deep water bodies. The second lake is notable for its greenish hue, which may be indicative of a high density of aquatic vegetation or the presence of algae. Both are surrounded by a mountainous environment with low vegetation cover. The NDWI method with Otsu thresholding faces challenges when segmenting lakes with a high density of aquatic vegetation, yielding negatively compromised segmentation results. In contrast, WatNet, DeepWaterMapV2, and WaterSegDiff demonstrate visually better segmentation, with WaterSegDiff standing out as the method achieving the best result in the context of lakes with aquatic vegetation.

In Figure 10c, two lakes are visible: the first one, situated at the top, displays an irregular yet well-defined shape, highlighted by its dark color contrasting with the surroundings. The second lake, located at the bottom, exhibits a greenish hue. Moreover, the presence of a river traversing the scene is noticeable, accompanied by scattered green areas. The segmentation performed by the different methodologies is generally accurate. However, the NDWI methodology incorrectly predicts some pixels originating from the river. On the other hand, WatNet tends to underestimate the area of small lakes, while DeepWaterMapV2 tends to overestimate it. In contrast to the other trained models, in this context, WaterSegDiff demonstrates a superior result, closer to the ground truth.

Figure 10d shows a large lake with a compact structure, containing a small, partially submerged piece of land. The edges of the lake are not clearly defined. In addition, light aquatic vegetation is observed. The scene contains mountainous regions that generate shadows. In this context, the trained models achieve excellent results in segmentation. However, the NDWI methodology has difficulties to accurately detect the edges of the lake and areas with aquatic vegetation. On the other hand, despite the presence of large shadows cast by the surroundings, the models do not have difficulty distinguishing them from the lakes.

Figure 10e depicts a lake situated in a mountainous region, surrounded by dense vegetation on its margins. This makes it challenging to discern the lake even with the unaided eye. In this complex context, both the NDWI methodology and the WatNet model struggle with accurately segmenting the lake’s edges. In contrast, DeepWaterMapV2 achieves the most precise segmentation, generating compact and well-defined contours. WaterSegDiff also yielded satisfactory results in this context.

In high-mountain ecosystems, snowy areas host lakes of various sizes and shapes. Figure 11a illustrates a mountainous landscape with snow and shadows created by the terrain’s irregularities. The scene contains both clear and turbid lakes, or lakes with sediments. The NDWI methodology with Otsu thresholding correctly segments the lakes; however, it also produces inaccurate segmentations, confusing shadow and snow areas with water bodies. In contrast, trained models offer more accurate results by avoiding these confusions. In this context, the WatNet model achieved the segmentation closest to the ground truth, accurately segmenting both clear and turbid lakes.

In Figure 11b, a complex environment is observed, characterized by extensive shadows and scattered snowy areas across the scene. Additionally, a brown-toned lake is discernible, along with other smaller bodies of water. In this context, it is noteworthy that the WatNet model achieved the highest segmentation quality by identifying the turbid lake, whereas comparative models such as DeepWaterMapV2 and WaterSegDiff failed to detect this lake.

Figure 11c depicts an environment that is almost entirely covered in snow, with lakes situated within the snow structure. A sizable lake is discernible in the scene, situated alongside smaller lakes that are dispersed throughout the area. Despite the scene’s complexity, all models demonstrated the capability to accurately distinguish the largest lake and avoid confusing shadows with bodies of water. However, the WatNet model is less effective in segmenting small lakes in this context. Conversely, both DeepWaterMapV2 and WaterSegDiff exhibited superior segmentation quality, accurately detecting small lakes.

In Figure 11d, a scene partially covered in snow is observed, with extensive shadows cast by mountain ranges. The lakes are located near the snow-covered areas, as well as within the mountain ranges. In this context, the NDWI methodology tends to confuse shadows with lakes. On the other hand, the trained models achieve accurate segmentations for the larger lakes near the snow-covered areas. WatNet has difficulties segmenting small lakes, whereas DeepWaterMapV2 provides the best segmentation for this scene.

In Figure 11e, the lakes are situated near mountainous formations, with the surrounding area partially covered by vegetation. A large lake is observed alongside others of varying sizes distributed throughout the scene. In this context, the segmentation performed by the WatNet model was inadequate, especially for the largest lake, although it accurately detected medium-sized lakes. The smaller lakes could not be detected by this model. Instead, WaterSegDiff successfully segmented the large lake but struggled to detect the small lakes near the mountains. The DeepWaterMapV2 model achieved the highest segmentation quality despite the scene’s complexity.

### 4.3. Temporal Analysis of Lake Singrenacocha

A temporal analysis of Lake Singrenacocha (located at 13°39′37.562″ South, 71°9′30.456″ West) was conducted. This popular tourist destination is nestled in the Vilcanota Mountains near Cuzco city, Peru. To further evaluate and understand the impact of environmental conditions, an analysis was conducted using four samples taken in May and June from different years, 2014, 2016, 2018, and 2020. The samples were selected to mitigate the influence of atmospheric effects caused by the El Niño and La Niña phenomena, according to [65].

**Figure 12:**
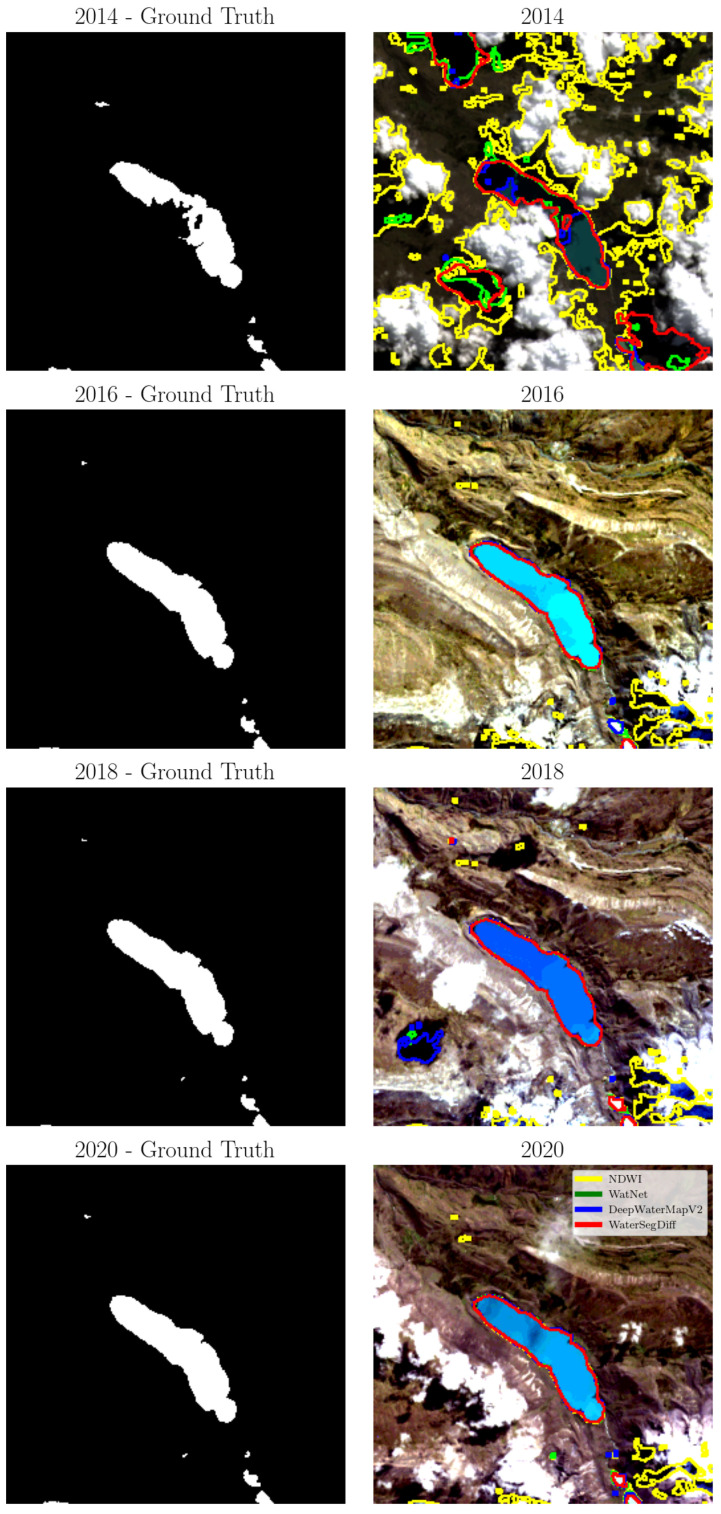
The edges extracted from Lake Singrenacocha based on NDWI, WatNet, DeepWaterMapV2, and WaterSegDiff. Highlights in yellow, green, blue, and red for the years 2014, 2016, 2018, and 2020, respectively.

The segmentation of Lake Singrenacocha was determined using the NDWI with Otsu’s method for automatic thresholding, as well as the WatNet, DeepWaterMapV2, and WaterSegDiff models. The boundaries of the segmented lakes were extracted using the Canny algorithm and are represented in yellow, green, blue, and red, corresponding to the NDWI, WatNet, DeepWaterMapV2, and WaterSegDiff models, respectively. The visual comparison of these results is shown in Figure 12, these images were acquired on the dates 6 May 2014, 12 June 2016, 18 June 2018, and 6 May 2020. In the figure, the ground truth is displayed on the left, and the edges obtained with the analyzed models are highlighted on the right.

**Figure 13:**
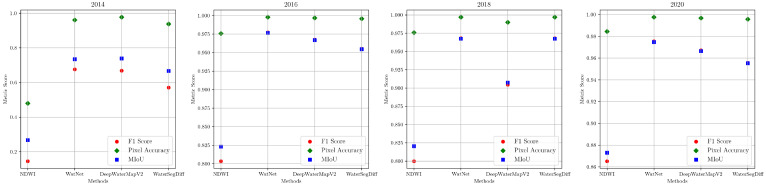
Graphical representation of the segmentation performance of Lake Singrenacocha during the years 2014, 2016, 2018, and 2020.

In addition to the visual evaluation obtained through temporal analysis, metrics were calculated for the four samples investigated, covering the years and methods analyzed. These metrics are detailed in Table 4 and graphically represented in Figure 13.

Regarding the temporal analysis of Lake Singrenacocha, a detailed examination of the 2014 sample, presented in Figure 12, reveals that the presence of clouds and shadows has a significant impact on the segmentation performance of all the models considered. In particular, the NDWI method has a notable decrease in accuracy due to the difficulty of distinguishing between clouds and lake features. In contrast, WatNet appears less affected by cloud shadows, although it predicts some as if they were lakes, showing discrepancies when compared to the ground truth. DeepWaterMapV2 and WaterSegDiff are also affected by cloud shadows but achieve results that are closer to reality. This analysis is confirmed by the metrics shown in Table 4, where the NDWI obtained values below 0.5, while WatNet and DeepWaterMapV2 showed similar values, with WaterSegDiff demonstrating competitive performance among the techniques. The graphic comparison can be visualized in the Figure 13.

In the 2016 sample, Figure 12, the NDWI method classifies mountain shadows as lakes, diverging from the ground truth, while WatNet, DeepWaterMapV2, and WaterSegDiff provide predictions much closer to the actual lake boundaries. Low cloud cover in 2016 allows these models to better distinguish between lake and mountain features, resulting in more accurate segmentation. Examining the metrics for the 2016 sample in Table 4, it is observed an improvement in all the methods analyzed compared to the 2014 sample, indicating how the presence of clouds affects segmentation. However, once again, the NDWI had the worst performance among the methods analyzed, while WatNet achieved the best metrics, which can be verified in the graphical comparison shown in Figure 13.

For the 2018 sample shown in Figure 12, the approaches again encounter problems with cloud shadows. However, since the clouds are not directly over the lake as in the 2014 sample, the behavior of the models in the segmentation task is relatively different. The NDWI method shows an improvement compared to 2014, which also features clouds. On the other hand, WatNet and DeepWaterMapV2 show substantial improvements, although with variable behaviors. For example, while WatNet generally exhibits improved performance, DeepWaterMapV2 mistakes a cloud shadow for a lake, as seen in the lower left of the image. Thus, for the 2018 sample, the best behavior is achieved by the WaterSegDiff model. Analyzing the metrics for 2018 sample in Table 4, NDWI once again has the worst metrics. However, this time, DeepWaterMapV2 also performed poorly compared to WatNet and WaterSegDiff, which behaved very similarly to each other, verified in the graphical comparison shown in Figure 13.

A closer look at the 2020 sample shows moderate cloud cover, but there was no confusion on the part of the models, resulting in an accurate segmentation of the lakes. This may have occurred because the shadows are not as pronounced as those seen in the 2018 sample, allowing the models to approximate the actual edge of the lake observed in the ground truth image. Among all the models analyzed, NDWI once again exhibits the worst performance, while WatNet, DeepWaterMapV2, and WaterSegDiff show similar behavior and produce adequate results. Finally, in the analysis of the 2020 sample metrics shown in Table 4, we observed poor performance by the NDWI, with WatNet once again demonstrating the best performance as show in Figure 13.

From the analysis from the samples in the years 2014 and 2018, illustrated in Figure 12, it is evident that mountainous terrain, especially when covered by a dense layer of clouds and shadows, can significantly complicate segmentation, leading to errors in lake edge extraction. This phenomenon particularly affects the NDWI method, which tends to produce inferior results due to the presence of clouds that make it difficult to differentiate statistically between lake and cloud characteristics. In fact, the NDWI method often overestimates areas of shadows and clouds, misclassifying them as lakes. In contrast, the predictions generated by the WatNet and DeepWaterMapV2 models are far more consistent and are less affected by the presence of clouds and shadows. This consistency is particularly evident in sampĺes with minimal or no cloud cover, such as 2016 and 2020, which tend to produce more reliable results in all approaches.

## 5. Discussion

This study evaluates the performance of different methodologies for lake segmentation in high-mountain Andean environments in Peru. For comparison, we employed the traditional NDWI method with Otsu thresholding, alongside three deep learning architectures: WatNet, DeepWaterMapV2, and WaterSegDiff. The choice of these architectures was based on their performance and the similar methodology used in each study. The WatNet and DeepWaterMapV2 models, based on CNNs, residual networks, and UNet-type networks, are fundamental options in image processing. Both methodologies utilize the capability of CNNs to gather global information and detect features at various scales through pooling and residual connections. However, CNNs overlook the spatial relationships between the underlying objects [66]. On the other hand, WaterSegDiff combines diffusion architectures and conditioning based on UNet, integrating this information through transformers that leverage their attention capabilities. This allows the model to obtain representations with a broader global context.

The results show that the NDWI methodology obtained the lowest values across all metrics. This can be explained by the nature of the images, which come from a high-mountain ecosystem where snow, clouds, and shadows generated by the geography have a reflectance similar to water, which is a known limitation in the segmentation of water bodies using this method.

The WatNet model, with metric values significantly outperforming the NDWI methodology, shows a higher Pixel Accuracy value than the MIoU value, which can be explained due to the class imbalance in the data and test set, with the “non-lake” class being the dominant one. This reflects that the WatNet model tends to classify correctly most of the pixels as “non-lake”, while the MIoU, by considering the overlap between the prediction and the ground truth, is more sensitive to errors in the segmentation of the lakes.

DeepWaterMapV2, which achieved the best results in the metrics, shows high accuracy in pixel classification, suggesting a notable ability to correctly identify both lake and non-lake classes. Its F1 Score of 0.8801 indicates an optimal balance between precision and recall, meaning that the model effectively captures both lakes and non-lakes in the image, minimizing both false positives and false negatives. The MIoU of 0.9088 reflects adequate overlap between the model’s predictions and the ground truth, indicating good quality in lake segmentation.

WaterSegDiff, although showing competitive performance, is inferior compared to DeepWaterMapV2 and WatNet. Despite having significantly higher complexity with 129.4 million parameters, it did not surpass these two models. According to the metrics in Table 3, DeepWaterMapV2 demonstrated the best results, closely followed by WatNet, with values very similar to each other. It should be noted DeepWaterMapV2 has 37.2 million parameters, while WatNet only has 3.7 million. Despite this significant difference in model complexity, both achieved comparable results, with DeepWaterMapV2 standing out slightly over WatNet. The analysis of metrics reveals that segmentation using the diffusion model (WaterSegDiff) does not surpass that achieved by the encoder–decoder architectures (WatNet and DeepWaterMapV2). This discrepancy may stem from differences in the training and testing characteristics of the models. While diffusion models learn incrementally, encoder–decoder networks learn over epochs, potentially leading to an imbalanced comparison.

Furthermore, when examining the F1 Score results in Table 3, it can be seen that the highest value achieved was less than 0.9, which may be due to the use of Landsat TOA products, which have a time difference of at least eleven days in a typical product creation period [67]. This time difference means that there is overlap between adjacent image scenes due to changes in atmospheric and lighting conditions. Another possible cause of this time difference is the multispectral characteristics of the images obtained by the Landsat-8 scenes; although they were acquired on the same day, the data show temporal differences for each band acquired. In addition, the data show variations in pixel values within the overlapping regions of each image due to different viewing angles and shadows in the mountainous terrain around the lakes.

In the qualitative evaluation of lake segmentation techniques in high-mountain environments, various distinctive characteristics were observed among the evaluated models. Overall, all methodologies performed well in segmenting deep lakes with contrasting features compared to their surroundings. However, the NDWI index with Otsu thresholding showed significant limitations in detecting lakes with a greenish hue due to the presence of algae or aquatic vegetation, as well as in identifying small water bodies and confusing shadows and snow with water bodies. In contrast, trained models such as WatNet, DeepWaterMapV2, and WaterSegDiff proved to be more robust in these contexts.

DeepWaterMapV2 excelled in the precise segmentation of both small and large water bodies, leveraging its FCN and UNet-based architecture to capture features at different scales. WatNet demonstrated a remarkable ability to detect lakes of various sizes and turbid lakes, although it struggled with the segmentation of small lakes due to the lower resolution in its MobileNetV2-based encoding layers. WaterSegDiff, on the other hand, showed promising results in segmenting lakes in complex contexts, though its performance could benefit from further hyperparameter optimization and pre-training techniques in unsupervised learning tasks followed by transfer learning.

The temporal analysis of Lake Singrenacocha highlight the robustness of the models for segmenting lakes in complex contexts, such as the mountainous environments of Peru, where there are cloud cover and shadows. Comparing the performance in the temporal analysis with the results obtained in Section 4.2.2, it is observed that the results are consistent and follow the same behavior. DeepWaterMapV2 and WatNet achieve the best segmentations, while WaterSegDiff shows promising results, with all models outperforming the segmentation methodology using the NDWI with Otsu thresholding.

These results highlight the importance of considering the variability of high-mountain environments and the specific challenges associated with segmenting water bodies in these regions. WatNet and DeepWaterMapV2 were the most effective models in this context, demonstrating greater robustness in complex conditions. The continuous optimization and adaptation of these models remain crucial areas for future research, aiming to further improve their accuracy and robustness.

## 6. Conclusions

This study explores lake segmentation in the Ancash and Cusco regions of Peru using high-resolution Landsat-8 imagery. Comparison between the NDWI with Otsu, WatNet, DeepWateMapV2, and WaterSegDiff showed that DeepWaterMapV2 achieved the highest overall performance (MIoU: 0.9088, PA: 0.9967, F1: 0.8801). WatNet also delivered strong results (MIoU: 0.9016, PA: 0.9960, F1: 0.8725) with a smaller model size, making it suitable for resource-limited scenarios. Although WaterSegDiff had lower performance, it remains a viable alternative. Qualitative analysis confirmed the superior segmentation capabilities of WatNet and DeepWaterMapV2 over the traditional method using the NDWI. Additionally, a temporal analysis of Lake Singrenacocha in the Vilcanota Mountains was carried out. Images were captured specifically in May and June of 2014, 2016, 2018, and 2020 to minimize the influence of the El Niño and La Niña phenomena. The findings highlighted WatNet’s effectiveness in detecting lakes even under adverse environmental conditions over time.

The results demonstrate that deep learning techniques analyzed for lake segmentation in high-mountain environments in Peru can significantly improve the accuracy and efficiency of ecological inventories. This has important implications for environmental monitoring and management, particularly for government institutions responsible for the sustainability of these ecosystems. Adopting these methods could reduce common human errors in traditional techniques such as the NDWI, thus optimizing conservation and environmental management efforts in the region.

In future work, there is an interest in using a larger amount of data to train the models for temporal analysis in order to verify the impact of climate change on water resources. In addition, to enhance the semantic segmentation task, several strategies can be implemented, such as employing hyperparameter optimization techniques to maximize the model’s performance and optimizing the WaterSegdiff diffusion model, which uses transformers, to compete with lighter models and scale on systems with fewer computational resources. Additionally, considering Landsat Collection 2 Level-2 data, which undergoes atmospheric correction for precise surface reflectance measurements, and leveraging Sentinel-2 Level-2A satellite imagery, which also offers atmospheric correction, could significantly enhance the segmentation process. Integrating multispectral data fusion with these datasets could further augment the model’s information input, leading to improved segmentation accuracy.

## Figures and Tables

**Table 1:** Landsat-8 Bands utilized for dataset creation [52].

Bands	Name	Wavelength
Band 2 (B2)	Blue	0.450–0.51 µm
Band 3 (B3)	Green	0.53–0.59 µm
Band 4 (B4)	Red	0.64–0.67 µm
Band 5 (B5)	Near-Infrared	0.85–0.88 µm
Band 6 (B6)	SWIR 1	1.57–1.65 µm
Band 7 (B7)	SWIR 2	2.11–2.29 µm

**Table 2:** Data split summary.

Category	Number	Resolution
Train	3228	256 × 256
Val	402	256 × 256
Test	402	256 × 256

**Table 3:** Standard metrics and parameters for quantitative analysis of the models.

Method	MIoU	PA	F1 Score	Parameters (M)
NDWI	0.6437	0.8034	0.5395	-
WatNet	0.9016	0.9960	0.8725	3.4
DeepWaterMapV2	0.9088	0.9967	0.8801	37.2
WaterSegdiff	0.8243	0.9895	0.7504	129.4

**Table 4:** Comparison of segmentation performance of Lake Singrenacocha during the years 2014, 2016, 2018, and 2020.

Year	Metrics	NDWI	Watnet	DeepWaterMapV2	WaterSegDiff
2014	MIoU	0.2670	0.7346	0.7391	0.6667
	PA	0.4797	0.9606	0.9770	0.9375
	F1 Score	0.1452	0.6758	0.6683	0.5700
2016	MIoU	0.8229	0.9766	0.9667	0.9547
	PA	0.9757	0.9977	0.9967	0.9956
	F1 Score	0.8034	0.9773	0.9674	0.9551
2018	MIoU	0.8205	0.9674	0.9074	0.9674
	PA	0.9757	0.9968	0.9899	0.9969
	F1 Score	0.7998	0.9681	0.9044	0.9681
2020	MIoU	0.8731	0.9747	0.9664	0.9553
	PA	0.9845	0.9975	0.9967	0.9957
	F1 Score	0.8652	0.9754	0.9671	0.9557

## Data Availability

The data supporting the conclusions of this article will be made available by the authors on request.
